# Esthetic and Functional Outcomes of Superficial Parotidectomy Comparing Three Reconstruction Techniques: An Interventional Clinical Study

**DOI:** 10.1055/s-0044-1788911

**Published:** 2025-04-22

**Authors:** Sherif Mohammad Askar, Abd ElRaof Said Mohamed, Tamer Oraby, Ibrahim Khaled, Mahmoud Megahed, Ali Awad

**Affiliations:** 1 Department of Otorhinolaryngology–Head and Neck Surgery, Faculty of Medicine, Zagazig University, Zagazig, Sharkia Governorate, Egypt

**Keywords:** superficial parotidectomy, sternomastoid muscle flap, fat graft

## Abstract

**Introduction** Preauricular defect is one of the main concerns after superficial parotidectomy. Plastic surgeons have described many filling techniques to overcome the problem.

**Objective** To discuss three reconstruction techniques after superficial parotidectomy: partial-thickness, superiorly based sternocleidomastoid muscle flap; en-bloc fat graft; and platelet-rich fibrin gel, with a comparison of aesthetic and functional outcomes.

**Methods** The present study included 29 adult patients submitted to reconstruction after superficial parotidectomy by partial-thickness, superiorly based sternocleidomastoid muscle flap, en-bloc fat graft, and platelet-rich fibrin gel. A subjective evaluation of the facial nerve functions was conducted through a visual analog scale (VAS) with scores from 0 to 5, which was completed by the patient, a close relative, and 3 blinded staff members.

**Results** Regarding the VAS, in the comparison of the 3 groups at the sixth and twelfth postoperative months, the fat-graft group reported the highest mean values for satisfaction (3.4 ± 1.1 and 3.83 ± 0.97 respectively). The fat-graft group also showed highly significant differences when compared with the groups submitted to the sternocleidomastoid muscle flap ( *p* = 0.0001) and the platelet-rich fibrin gel techniques ( *p* = 0.016).

**Conclusion** Parotidectomy with immediate reconstruction of the surgical defect through an en-block fat graft provides better esthetic outcomes than sternocleidomastoid muscle flap and platelet-rich fibrin gel after one year. The patients submitted to the sternocleidomastoid muscle flap and fat-graft techniques reported minimal surgical site morbidity and a lower chance of developing Frey syndrome. The fat graft resulted in the best degree of cosmetic satisfaction, with minimal morbidity. Fat overcorrection is recommended.

## Introduction

The most common indication for superficial parotidectomy (SP) is the removal of a benign neoplasm; 75% to 80% of neoplasms of the parotid gland are benign. Pleomorphic adenoma and Warthin tumor account for most of the tumors encountered. 1 The main target of SP is the removal of the entire benign tumor (with its capsule) without injuring the facial nerve. Surgical hazards include Frey syndrome (FS) and numbness of the ear lobule. Several esthetic issues might follow SP, and facial asymmetry, scar deformity, and hollowed-out preauricular defects are the main concerns. Modern surgeons tend to deal with these ordeals through modifications in the classic incision, local flaps, and volumetric autogenous soft tissue transfer to fill the surgical defect. 2 3 4 5 6 7 After a review of the available English literature, we found that to date there is no consensus among surgeons regarding the best technique. Moreover, the optimum protocol is still controversial.

Partial-thickness sternocleidomastoid muscle flap (SCF) features among the various methods described to correct postparotidectomy defects, and it presents low rates of donor-site morbidity. 4 5 8 On the other hand, primary, satisfactory esthetic outcomes have been reported with fat grafts. 9 10 Platelet-derived growth factors can stimulate tissue repair, support cellular proliferation and differentiation, and influence extracellular matrix deposition. Thus, platelet-rich fibrin (PRF) gel has been used in different surgical indications aiming at improving outcomes. 11 12 13 14 15 16 17 18

The goal of the present work was to study three popular postsuperficial parotidectomy reconstruction techniques: SCF, en-bloc fat graft, and PRF, with a comparison of the esthetic and functional outcomes.

## Methods

### Settings

The present prospective, randomized, interventional clinical study was conducted at the Department of Otorhinolaryngology–Head and Neck Surgery of the Faculty of Medicine of Zagazig University, Egypt, from May 2018 to March 2023.

### Reporting Guidelines

Specific guidelines about the esthetic outcomes after parotidectomy are lacking; therefore, we followed the clinical practice guidelines of the American Society of Clinical Oncology (ASCO). 19

### Inclusion and Exclusion Criteria

The current study included adult patients who underwent SP for benign parotid lesions. Patients with malignant pathology, recurrent parotid lesions, and previous radiotherapy were excluded. The follow-up period ranged from 13 to 20 (mean: 15 ± 1.07) months.

### Methods

After preoperative preparation, the patients underwent SP with immediate reconstruction. A modified Blair incision was used in all patients, and the sample was randomly stratified into 3 subgroups according to the reconstruction technique: group 1–SCF; group 2–fat graft; and group 3–PRF. The operative time was defined as the time until the completion of the reconstruction method; the time it took to perform the SP and parotidectomy wound closure was not considered.

#### Surgical Technique for Superficial Parotidectomy

The patients were positioned in the usual supine position with their necks tilted for SP, the incision began as a standard preauricular curvilinear incision in front of the tragus, moving downwards around the inferior border of the lobule, the tip of the mastoid process, and then forward in the skin crease. The skin flaps were elevated, and the greater auricular nerve was identified and dissected. The facial nerve trunk was identified by the standard anatomic landmarks. 1 2 The procedure was accomplished with the dissection and preservation of facial nerve branches from underlying parotid tissue.

##### Assessment of the Specimen Volume

The resected tissues were submerged in a scaled specimen cup filled with distilled water, and the overflowing volume of the water was defined as the specimen's volume (SV).

##### Partial-thickness, Superiorly based Sternomastoid Flap

**Fig. 1:**
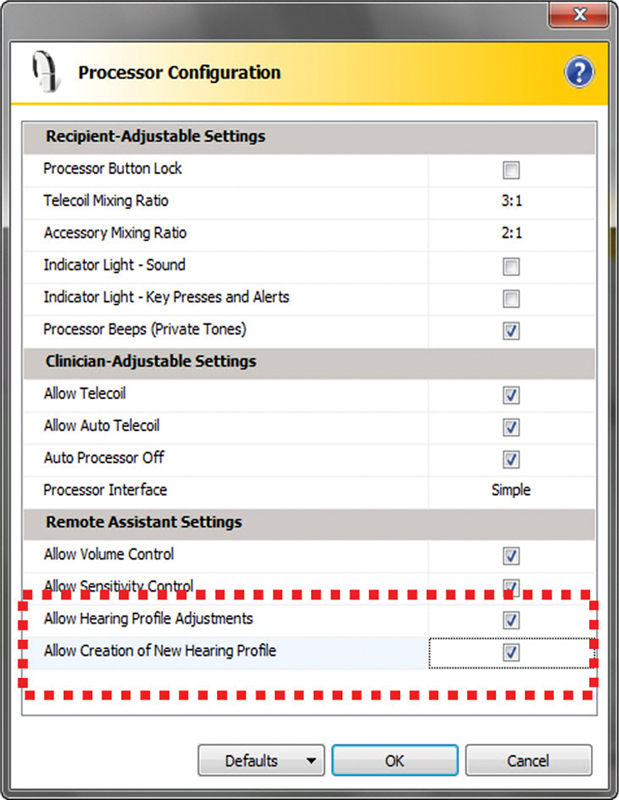
Partial-thickness, superiorly based sternocleidomastoid muscle flap.

The length of the SCF was first estimated by measuring the length of the defect; the thickness of the flap varied according to the size of the gap. The SCF was elevated and rotated anteriorly to be sutured to the parotid fascia. This would fill the surgical defect and cover the facial nerve (and its branches), the retromandibular vein, and the external carotid artery (and its terminal branches). Then, a drain was placed deep into the flap ( Fig. 1 ).

#### Fat-graft (En-bloc Fat) Technique

**Fig. 2:**
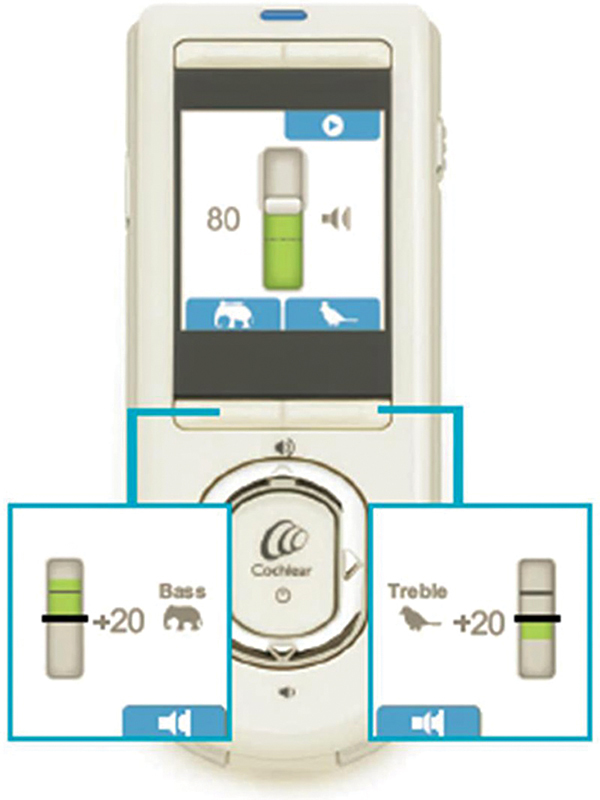
The fat-graft in place.

A periumbilical incision was performed. The fat graft volume was assessed using the submerging technique in a scaled cup. The fat (en-bloc) graft was placed in the surgical defect and sutured to the remaining parotid tissue (with VICRYL 3-0 sutures, Ethicon, Inc., Raritan, NJ, United States) to ensure graft fixation. The operative time to harvest the fat graft and close the abdominal incision was calculated, blood loss from the donor site was recorded, and non-suction rubber drains were used ( Fig. 2 ).

#### Platelet-rich Fibrin Gel

**Fig. 3:**
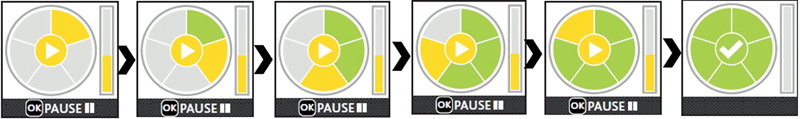
The platelet-rich fibrin gel in place.

For PRF preparation, a peripheral venous blood sample was drawn from the patient and transferred to 10-mL sterile tubes (with no anticoagulants nor calcium). The tubes were centrifuged (model 800, Jiangsu Zhengji Instruments Co., Ltd., Jintan City, China) for 10 minutes at 3,200 rpm. The blood would show three layers: the bottom layer (of red blood cells), the middle layer (of PRF; platelets, and white blood cells), and the top layer (of platelet-poor plasma). The PRF layer (50–60% of the blood in the tube) was extracted before application (30–40 mL) and was then put on a sterile gauze pad to absorb the serum ( Fig. 3 ). 18

##### Volume Assessment and Wound Closure

The volumes of the fat graft and PRF were estimated as 40% to 50% higher than the SV. The wound was closed in layers over a non-suction drain.

#### Postoperative Follow-up

The drains in the parotid incisions (and abdomen) were removed when a minimal collection was noticed. Examinations of the face and donor sites (abdomen) and functional outcomes were performed weekly for the first month, then monthly for 3 months, and then every 3 months until the end of the follow-up sessions. The functional evaluation included FS (starch iodine test 8 13 ), facial nerve integrity, and ear lobule sensation. The esthetic evaluation included facial deformity (preauricular/retromandibular defects) and the scar incision. The subjective evaluation of the facial nerve functions was performed using a visual analog scale (VAS-f scale; Table 1 ); this newly-designed scale with scores from 0 to 5 points was completed by the patient, a close relative, and 3 blinded surgeons, and the scores are as follows: 5–normal appearance, symmetrical to the opposite side; 4–minimal asymmetry, barely noticeable from a short distance; 3–mild asymmetry, noticeable but with no disfigurement; 2–moderate asymmetry, mainly in the preauricular area; 1–severe asymmetry, with deep preauricular/retromandibular groove (PRG); and 0–severe asymmetry, with deep PRG and obvious scar. And the results collected from the 5 personnel (the patient, a close relative and three surgeons) were as follows: 0 to10–poor; 11 to 15 – fair; 16 to 20 – good; and > 20–excellent.

**Table 1:** The designed Visual Analog Scale (VAS-f scale)

Degree	Appearance
5	Normal appearance, symmetrical to the opposite side
4	Minimal asymmetry, barely noticeable from a short distance
3	Mild asymmetry, noticeable but with no disfigurement
2	Moderate asymmetry, mainly in the preauricular area, is apparent
1	Severe asymmetry, with a deep preauricular and retromandibular groove
0	Severe asymmetry, with a deep preauricular, retromandibular groove, and an obvious scar

### Statistical Analysis

All data were collected, tabulated, and statistically analyzed using the IBM SPSS Statistics for Windows (IBM Corp., Armonk, NY, United States) software, version 23.0. The quantitative data were expressed as mean ± standard deviation (SD) and range values, and the qualitative data, as numbers and percentages. The analysis of variance (ANOVA) test was used to compare more than two groups of normally distributed variables. The paired *t* -test was used to compare pairs of normally distributed variables. The *p* -values were considered statistically significant if < 0.05 and highly significant if < 0.001.

## Results

### Patient Characteristics

The present study included 29 (17 female and 12 male) patients with a mean age of 50.48 ± 9.1 (range: 33–66) years, who were randomly distributed into 3 subgroups: group 1 included 11 (8 female and 3 male) patients submitted to SCF; group 2 included 9 (6 female and 3 male) patients who underwent fat graft; and group 3 included 9 (7 female and 2 male) patients submitted to the PRF technique. The 29 patients included presented unilateral benign parotid swellings for more than 6 months, and pleomorphic adenoma was the most common tumor mass encountered (19 patients, 65.52%). The size of the tumors ranged from 14 to 30 (mean: 21.4 ± 4.7) cm ^**3**^ .

### Outcomes

Fat harvesting with wound closure was accomplished during the last steps of SP within 15 to 22 (mean: 18 ± 1.35) minutes. Blood loss from the periumbilical incision ranged from 10 to20 (mean: 14.4 ± 4.6) mL. There were no cases of postoperative fat necrosis (drainage of fat from the parotidectomy incision for a few days). The SCF was performed after finishing the SP; the duration of flap harvesting and suturing was of 9 to 14 (mean: 11 ± 1.05) minutes.

There were 6 cases (20.69%) of FS: 3 (33.3%) patients from the fat-graft group, 2 (22.2%) patients from the PRF group, and 1 (9.1%) patient from the SCF group. By 12 months, 1 patient in the PRF group was still complaining of gustatory sweating.

Neck stiffness was reported in 2 (18.2%) male patients from the SCF group; it improved after 3 to 6 months. Ear lobe numbness was reported by 6 patients (3 from the SCF, 1 from the fat-graft, and 2 from the PRF groups) which improved within 8 to 12 weeks. In the fat-graft group, abdominal pain was reported by 2 patients (22.2%); the pain disappeared after 3 to 4 weeks. Abdominal seroma was reported by 1 patient (11.1%); conservative management was effective. At 6 months, no patients reported any problems regarding the abdominal incision. A comparison of the number of postoperative complications in the 3 groups showed that nonsignificant results were obtained ( *p* = 0.407; Chi-squared =1.7).

According to the VAS-f scores, after 6 months, the SCF group reported fair results in 3 patients and good results in 8 patients; the fat-graft group reported good results in 5 patients and excellent results in 4 patients; and the PRF group reported fair results in all 9 patients. After 12 months, the SCF group reported good results in 9 patients and excellent results in 2 patients; the fat-graft group reported good results in 2 patients and excellent results in 7 patients; and the PRF group reported fair results in 5 patients and good results in 4 patients.

**Fig. 4:**
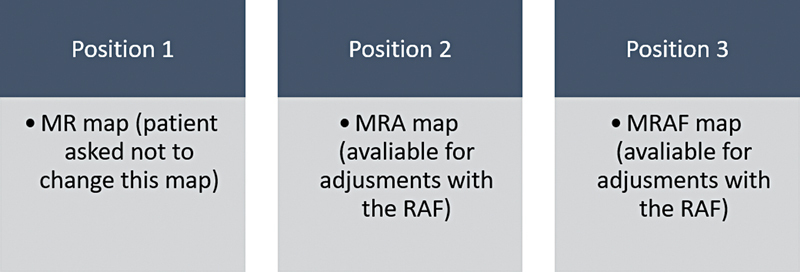
Mean scores on the Visual Analog Scale (VAS-f) at 6 and 12 months postoperatively for the three groups according to different evaluators.

Regarding the level of satisfaction, in the comparison of the three groups (after 6 and 12 months), the fat-graft group reported the highest mean values (of 3.4 ± 1.1 and 3.83 ± 0.97, respectively). The fat-graft group also showed a highly-significant difference when compared with the SCF and PRF groups ( *p* = 0.0001 and 0.016 respectively). The comparison of individual patient satisfaction at 6 and 12 months was highly significant in the SCF group (t = 5.2; *p* = 0.0001) and in the PRF group (t = 5.3; *p* = 0.001), while it was nonsignificant in the fat-graft group (t = 1.2; *p* = 0.28). According to the opinion of experts, the fat-graft group reported the highest mean value for satisfaction at 6 and 12 months (of 4.2 ± 0.41 and 4.4 ± 0.46 respectively). The comparison of the fat-graft group with the PRF group revealed highly significant differences ( *p* = 0.0001), while the comparison of the fat-graft group with the SCF group revealed nonsignificant differences at 6 months ( *p* = 0.068), but significant differences at 12 month ( *p* = 0.03). The comparison of the experts' opinions at 6 and 12 months was significant in the fat-graft group (t = 4; *p* = 0.001) and the PRF group (t = 5.3; *p* = 0.001), while it was nonsignificant in the SCF group (t = 1.84; *p* = 0.096) ( Table 2 ; Fig. 4 ).

**Table 2:** Postoperative VAS-f score at 6 and 12 months

Variables	Study groups (N = 29)			f	*p*	Post-hoc test
	SCF	PRF	Fat graft			
Cosmetic satisfaction of patients at 6 months	1.6 ± 1.1	2.2 ± 0.7	3.4 ± 1.1	8.1	0.002	(0.21)* (0.0001)** (0.016)***
Cosmetic satisfaction of patients at 12 months	3.3 ± 0.9	3 ± 0.87	3.8 ± 0.97	1.7	0.205	−
Paired t; P1	5.2; 0.0001	5.3; 0.001	1.2; 0.28			
Cosmetic satisfaction of relatives at 6 months	2.6 ± 0.67	2.8 ± 0.7	3.9 ± 1.2	6.1	0.007	(0.72)* (0.003)** (0.01)***
Cosmetic satisfaction of relativeS at 12 months	3.9 ± 0.54	3.4 ± 0.88	4.2 ± 1.2	1.7	0.196	−
Paired t; P1	6.5 0.0001	4 0.004	1.4 0.19			
Experts score at 6 months	3.8 ± 0.37	2.7 ± 0.5	4.2 ± 0.41	31.1	0.0001	(0.0001)* (0.068)** 0.0001)***
Experts score 12 months	4 ± 0.35	2.9 ± 0.46	4.4 ± 0.46	29.2	0.0001	(0.0001)* (0.03)** (0.0001)***
Paired t; P1	1.84 0.096	5.3 0.001	4 0.001			
Total score at 6 months	3.1 ± 0.14	2.6 ± 0.33	3.97 ± 0.35	46.2	0.0001	(0001)* (0.0001)** 0.0001)***
Total score at 12 months	3.8 ± 0.34	3 ± 0.37	4.2 ± 0.31	28.5	0.0001	(0.0001)* (0.01)** (0.0001)***
Paired t; P1	6.3; 0.0001	10; 0.0001	4; 0.004			

**Abbreviations:** f, analysis of variance (ANOVA); PRF, platelet-rich fibrin gel; SCF, partial-thickness sternocleidomastoid muscle flap; VAS-f, Visual Analog Scale.

**Notes:** Data: expressed as mean ± standard deviation values; P: comparison among the three groups; P1: comparison within each group; * *p* -values for the comparison between the SCF and fat-graft groups; ** *p* -values for the comparison between the PRF and fat-graft groups; *** *p* -values for the comparison between the SCF and PRF groups; *p* < 0.05: significant; *p* < 0.001: highly significant.

The comparison of each group at 6 and 12 months regarding sex revealed higher levels of satisfaction among male patients (in the three groups) than among female subjects. This difference was significant in the fat-graft and SCF groups ( *p* = 0.024 and 0.012 respectively at 6 months) and nonsignificant in the PRF group (at 6 months: *p* = 0.051; at 12 months: *p* = 0.064). The comparison of female and male patients in the same group showed significant differences in the SCF and in the fat-graft groups ( *p* = 0.012 and 0.024, respectively) and non-significant results in the PRF group ( *p* = 0.051) at 6 months; The same figures were obtained for the follow-up at 12 months ( *p* = 0.017, 0.0059, and 0.064, respectively).

## Discussion

Facial nerve injury is the eminent ordeal complication of SP; however, the most common hazards affecting the patients' quality of life are the evident cosmetic defects, the defective sensation of the ear lobule, and FS. 2 3 4 5 Surgeons have presented different options to overcome these problems: besides the well-planned parotidectomy incision, when applying these methods, they may employ a filler for the surgical defect, for it may act as a biological barrier between the skin and the parotid bed to prevent FS; some surgeons have tried SCF, other researchers have tried free fat grafts, and some have used PRF; free flaps have also been discussed. 2 7 9 10 12 20

The SCF technique has gained noticeable acceptance among surgeons. The flap is generous, easily harvested, lies within the surgical field, and yields a low risk of complications. It can be designed as superiorly- or inferiorly-based. 2 3 8 The immediate filling of the surgical defect with SCF yields good esthetic outcomes, with minimal donor-site morbidity. 2 3 4 5

Recently, the PRF technique has been used by different surgical specialities; it can be applied to fill cavities, as it contains several growth factors. Its basic components are fibrin matrix polymer, white blood cells, and blood aggregates, and it also contains stem cells. Thus, it can promote tissue regeneration and show adequate support for wound healing. Surgeons have applied PRF in SP and reported good outcomes. 11 12 13 14 15 21 22

Satisfactory esthetic outcomes have been reported for primary fat transfer after SP. However, the technique requires a separate incision site and may result in donor-site complications such as hematoma, fat necrosis, and infection. The exact determination of the fat volume needed for reconstruction in each patient is another obstacle. 9 10 20 Tunca et al. ^23^ (2021) studied the fat-graft resorption rates at 1 year and reported a mean of 50.75 ± 21.20%. 23 In the current study, we used the submersion technique to measure the volume of the excised specimen. At 6 months, the abdominal incision presented minimal morbidity.

In the present study, FS was reported in 6 patients during the early follow-up visits. This number improved to 2 patients after 12 months. The SCF group presented the best results regarding FS. Although the number of patients is limited, these results might support SCF as a guard against FS. 24 25

In the current study, we employed the VAS-f scale to assess satisfaction. The fat-graft group reported the highest mean values for satisfaction according to the patients themselves, their relatives, and the experts at 6 and 12 months. The lowest rate of satisfaction was reported in the PRF group, while the SCF group occupied the middle position. The comparison of the fat-graft group with the other two showed highly significant differences at both follow-up visits. The comparison of the mean level of satisfaction at 6 and 12 months in the SCF group was highly significant, and the PRF group reported significant results, while a nonsignificant result was obtained in the fat-graft group. Moreover, the results of the present study showed more satisfaction among male subjects than female patients.

## Limitations

The current study has limitations. Firstly, it was short-term study with a relatively small number of patients. Secondly, the report of esthetic aspects is hampered by subjectivity and inter-observer variability. We tried to control this variability by reporting the assessments made by the patients themselves, a close relative, and three independent examiners. Thirdly, there was a difference in volume replacement among the three reconstruction techniques, with the fat graft and PRF volumes based on the volume of the specimen; the SCF volume was dependent on an estimate by the surgical team. Moreover, the current study did not include a control group. Further prospective long-term studies with larger samples are needed.

## Conclusion

Parotidectomy with immediate reconstruction of the surgical defect with an en-block fat-graft provides better esthetic outcomes than SCF and PRF after 1 year. The SCF and fat-graft techniques resulted in minimal surgical-site morbidity and lower chances of developing FS. The fat-graft yielded the best degree of cosmetic satisfaction, with minimal morbidity. Therefore, fat overcorrection is recommended.
